# Introduction to Bayesian Mindsponge Framework analytics: An innovative method for social and psychological research

**DOI:** 10.1016/j.mex.2022.101808

**Published:** 2022-08-05

**Authors:** Minh-Hoang Nguyen, Viet-Phuong La, Tam-Tri Le, Quan-Hoang Vuong

**Affiliations:** a Centre for Interdisciplinary Social Research, Phenikaa University, Ha Dong District, Hanoi 100803, Viet Nam; b AISDL, Vuong & Associates, Dong Da, Hanoi 100000, Viet Nam

**Keywords:** Bayesian inference, Mindsponge mechanism, Information process, Social sciences, Psychological and behavioral sciences

## Abstract

The paper introduces Bayesian Mindsponge Framework (BMF) analytics, a new analytical tool for investigating socio, psychological, and behavioral phenomena. The strengths of this method derive from the combination of the mindsponge mechanism's conceptual formulation power and Bayesian analysis's inferential advantages. The BMF-based research procedure includes six main steps, in which the mindsponge-based conceptualization and model construction is the key step that makes the method unique. Therefore, we elaborate on the fundamental components and functions of the mindsponge mechanism and summarize them into five memorable principles so that other researchers can capitalize directly. An exemplary analysis was performed using a dataset of 3071 Vietnamese entrepreneurs’ decisiveness and perceptions of the likelihood of success/continuity to validate the method.

• *The paper provides five strong points of BMF analytics, originating from the good match between the mindsponge mechanism and Bayesian inference.*
• *The paper also provides a step-by-step procedure for conducting BMF-based research.*
• *The mindsponge mechanism's basic components and functions are elaborated and summarized into five core principles that can be applied directly for research conceptualization and model construction.*

## Graphical abstract

**Figure:**
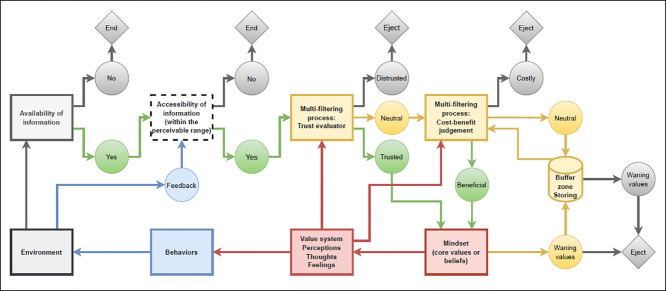

**Specifications table**

**Table:** 

Subject area:	Social Sciences; Psychology
More specific subject area:	*Information process* *Social psychology*
Name of your method:	*Bayesian Mindsponge Framework (BMF) analytics*
Name and reference of the original method:	Vuong Q-H, Nguyen M-H, & La V-P (2022) *The mindsponge and BMF analytics for innovative thinking in social sciences and humanities* (De Gruyter).
Resource availability:	The code and data supporting the exemplary analysis is available in the Supplementary

## Method details

In this article, we aim to introduce the Bayesian Mindsponge Framework (BMF) analytics, a novel method combining the strengths of the mindsponge mechanism and Bayesian inference for social and psychological investigations [1]. The current article's content is mainly extracted from the book: *The mindsponge and BMF analytics for innovative thinking in social sciences and humanities* [2].

### Strengths of BMF analytics

In the BMF, the mindsponge mechanism, with its ability to reflect the complexity and dynamics of a human mind, is used to construct theoretical models. At the same time, with its great flexibility, Bayesian inference allows researchers to fit those models. Moreover, the good match between the mindsponge mechanism and Bayesian inference produces five distinct strong points of BMF analytics. Thanks to these strengths, the BMF analytics has been successfully applied to explore various social, psychological, and behavioral issues in multiple disciplines, such as mental health [3], education [4], environmental psychology [5], public health [6], scientific publishing [7], security [8], etc.

Firstly, on a fundamental philosophical level, both Bayesian inference and mindsponge are based on subjectivity. From the mindsponge viewpoint, the mindset heavily influences one's perceptions, attitudes, and behaviors. These expressions as results of mental processes are individually and contextually specific. Subjectivity is exhibited in Bayes’ theorem, and Bayesian inference is built upon subjective probability. Completely avoiding subjectivity in psychological research can be considered impossible, and a method that can effectively deal with subjectivity is required [9]. The BMF analytics can help researchers account for subjectivity but do not compromise scientific rigor in both model conceptualization and statistical analysis.

Secondly, the BMF is highly flexible in investigating complex and dynamic processes of the human mind. The mindsponge mechanism can be used to construct models, including non-linear relationships that reflect the mind's information processes (see Section 2 for more details). Bayesian inference aided by Markov chain Monte Carlo (MCMC) algorithms allows for fitting high-dimensional and highly complex models [10], [11], [12]. In many cases, parsimonious models are preferable due to their explanatory predictive power. Regarding this aspect, Bayesian inference allows researchers to focus on estimating models of the targets of interest because it treats all properties (including unknown parameters and uncertainties) probabilistically. The mindsponge mechanism, constructed based on the set theory logic, provides a framework to define the boundary of studied subjects, which helps achieve parsimoniousness in model construction. The following empirical studies are typical examples of this strength of BMF analytics [4,7,8].

Thirdly, the BMF can effectively deal with hierarchical problems. The mindsponge mechanism helps construct models with parameters that reflect the differences in each level (e.g., individual and collective). Multilevel modeling is a suitable model for estimating variation across groups. Multilevel models are Bayesian in the sense of assigning probability distributions to the varying regression parameters [13], and MCMC techniques help overcome the complexity of multilevel modeling and compute posterior distributions [14]. Thus, Bayesian analysis aided by MCMC techniques can be used to fit complex hierarchical models containing nonlinearities [15].

Fourthly, while Bayesian inference's strength is the incorporation of prior knowledge, this is also a point of criticism. Although uninformative priors can be employed to avoid criticism of subjectivity [16], the benefits of performing Bayesian analysis can only be maximized when the researchers are capable of identifying the plausible informative priors. Using the mindsponge mechanism to logically construct models and justify prior distributions of parameters provides theoretical support and increases the precision of prior choices. If the prior is adequately specified, the Bayesian approach can provide more accurate inference for small sample datasets than the frequentist method [17]. With this, research activities’ costs can be adjusted well, supporting scientific endeavors in countries with limited funding and other resources for science [18]. Additionally, the incorporation of prior information helps solve the multicollinearity problem (correlation among independent variables causing less reliable statistical inference). Specifically, it has been found that the Bayesian approach with informative priors outperforms the Ridge regression approach [19,20].

Fifthly, both the mindsponge mechanism and Bayesian inference have the updating feature. This helps examine psychological phenomena on a temporal dimension in terms of information processes naturally going through the updating nature of the human mind and society (the infosphere). The ability to examine the effects of contextual factors on psychological processes can help with the current reproducibility crisis in psychology [21].

Besides the strengths derived from the good match of the mindsponge mechanism with Bayesian inference, the BMF analytics also inherit the theoretical advantages of Bayesian inference. Bayesian inference, with its basic features being estimation and visualization of credible intervals [15,22,23], has been advocated by many scientists as a good alternative to alleviate the ongoing reproducibility crisis in social sciences [24,25] and psychological research [26]. Bayesian inference employs credible intervals that treat estimated parameters as random variables and their bounds as fixed. In contrast, the frequentist approach employs confidence intervals that treat the estimated parameter as fixed and their bounds as random variables. Credible intervals help demonstrate regions where true parameter values have certain probabilities of being in. This distinction gives Bayesian inference more theoretical advantages compared to the frequentist approach [15].

The Bayesian approach provides a more precise estimation over small sample sizes compared to the frequentist approach, which saves the cost of investing in huge datasets. Bayesian inference does not make the distinction between unobserved data and unknown parameters and treats both known and unknown quantities probabilistically [22]. Due to this characteristic, Bayesian inference is not dependent on the asymptotic theory (or large sample theory), which assumes that the sample size may grow indefinitely, and the properties of estimators and tests are evaluated under the limit of n→∞. In other words, the estimated results from Bayesian inference are only conditional on the data at hand.

### The procedure for conducting BMF-based research

The BMF analytics is built on two fundamental principles: *effectiveness* and *efficiency. Effectiveness* refers to the significance (potential values of the research project) and the possibility of completing the research project (feasibility and thinkability). In contrast, *efficiency* refers to the notion of cost-effectiveness (the ratio between the benefit and cost of conducting the research project). When applying the BMF analytics, the mindsponge mechanism facilitates researchers’ thinking and imagination processes, improving the possibility of explaining complex and dynamic social and psychological phenomena. After ideas or postulations are successfully formulated, the Bayesian inference allows researchers to validate those ideas or postulations using small datasets, which substantially reduces the cost of doing science [18].

Conducting BMF-based research consists of six major phases. Although the mindsponge-based conceptualization and model construction and Bayesian analysis are two core steps of BMF analytics, the former is the key component that makes the method unique. As a result, in order to complete this vital step, researchers must first understand the mindsponge process. Here, we detail the key goals and components of each of the six phases. At the same time, the next section will primarily focus on explaining and summarizing components and functions of the mindsponge mechanism so that other researchers may readily utilize the mechanism in thinking and imaginative processes.

1) **Identify research question(s)/objective(s)**

The main objective of this step is to define the research objectives or questions that will be used to investigate the topics or phenomena of interest. During this step, researchers must also justify why the identified research questions/objectives are important by connecting them with sufficient and on-point background information. This contains an introduction to the topic under consideration and a review of pertinent literature. A good research question/objective is one that can guarantee effectiveness and efficiency throughout the scientific investigation process. In other words, a research question/objective that can lead to valuable scientific outcomes, maintain feasibility and thinkability, and only consume a minimal amount of resources is a good research question/objective.

2) **Conceptualize and construct a hypothetical model(s) based on the mindsponge mechanism**

The main objective of the second step is to employ the mindsponge mechanism to conceptualize and construct hypothetical models necessary to fulfill the research objective/question. This step does not have any fixed structure and constituents. It only requires researchers to understand the components and features of the mindsponge mechanism to utilize it effectively. For easing the comprehension of the mindsponge mechanism, detailed explanations of the mechanism are shown in the next section. In particular, the logical diagram presented in Fig. 2 can aid researchers in conceptualization, model construction, and even research question/objective identification.

3) **Design, collect, and process the data**

In this step, researchers prepare the data required to investigate the constructed models statistically. There are two major types of data. Apart from collecting primary data (the first-hand data that researchers collect by themselves), researchers can make use of secondary data (the data that are collected by others other than themselves). Considering research cost management, researchers can retrieve data from reliable open repositories, such as Figshare, Mendeley Data, Dryad Digital Repository, Harvard Dataverse, Open Science Framework, Zenodo, Science Data Bank, etc. In addition to these repositories, datasets published in data journals (e.g., *Scientific Data, Data in Brief, Data*) are also reliable resources for statistical analyses because they were systematically structured, validated, and peer-reviewed.

Regardless of generating a primary dataset or finding a secondary one, the dataset should acquire the following three elements to be considered qualified. First, the design and collection method of the dataset is rigorous. Second, the data are complete. In other words, the dataset's missing data should be minimal. Lastly, the observations should be diverse to ensure representativeness and avoid selection bias.

The most common data collection method in social sciences and humanities is a survey. This method is even more common in developing countries, where advanced technologies for scientific research are lacking. The most common type of survey data is discrete data. Given the complexity and dynamics of the mindsponge mechanism, one question arises: is it appropriate to use survey data for BMF analytics?

The answer is yes. To answer the questionnaire, survey respondents have to employ the existing information in their minds. In other words, they give the researchers their thoughts, feelings, and memory by answering the questionnaire. Although information perceived from the external environment when responding to the questionnaire can also affect the respondents’ answers, such effects are negligible most of the time. The mindsponge mechanism can help explain the psychological and behavioral processes that led to the thoughts, feelings, and memory that the respondents answered. In other words, the mindsponge mechanism enables an analytical framework that helps establish and imagine a psychological process retrospectively using the data at hand. The studies of Nguyen et al. [3] and Vuong et al. [4] are typical examples of this retrospective explanation.

4) **Perform the Bayesian analysis to test the model(s)**

The Bayesian analysis normally consists of five main steps.

Step 1: Constructing the models
Step 2: Fitting the models
Step 3: Diagnosing the models
Step 4: Interpreting the estimated results
Step 5: Comparing models

For performing Bayesian analysis, many software packages and programs are available and free to use, such as **WinBUGS** [27], **OpenBUGS** [28], **JAGS** [29], **MCMCglmm** [30], **Stan** [31], **brms** [32], **rethinking** [23], **rstanarm [****33****]**, and **bayesvl** [34]. Each software package or program has certain strong points compared to others, so researchers are recommended to study the operations and functions of each software package and program thoroughly to determine the one that suits their interest the most.

5) **Evaluate and report the observed results**

In this step, researchers diagnose, interpret, and present the estimated results, aligned with steps 3-5 of the Bayesian analysis mentioned above. For Bayesian analysis aided by MCMC techniques, it is crucial to check if the Markov chain central limit theorem is held. The convergence of Markov chains can be diagnosed by the effective sample size (*n_eff*) and Gelman shrink value (*Rhat*), as well as visualizing plots such as trace plots, Gelman plots, and autocorrelation plots. The Bayesian approach does not use the *p*-value to assess the hypothesis. Instead, we assess whether the hypothesized association between the predictor and outcome variables is reliable or not by assessing the parameters’ posterior distributions. Such results can be visualized using density plots, interval plots, distribution plots, two-dimensional density plots, etc.

6) **Discuss the observed results**

The last step is aligned with the discussion section in a research paper, where findings are connected and compared to related theories and other existing studies. Mindsponge-based reasoning can be used to effectively explain the similarities or distinctions between obtained results and the proposed conceptual models. Based on the study's findings and surrounding arguments, implications for policymaking and further studies should be presented. Last but not least, the limitations of the study should be clearly stated to uphold research transparency [35].

## Comprehending the mindsponge mechanism

Within the BMF analytics, mindsponge-based conceptualization and model construction is the key step that makes the method unique. Therefore, utilizing the method fluently requires researchers to understand the core principles of the mindsponge mechanism. We dedicate this section to elaborate on the basic components and functions of the mindsponge mechanism and summarize them into five memorable principles so that researchers can capitalize directly to conceptualize research and construct models. A logical diagram demonstrating the information process of the mechanism is also provided to aid readers’ comprehension.

The term mindsponge refers to the metaphor that the mind is analogized to a sponge that absorbs new compatible values and squeezes out incompatible values with its core values. Incorporating the existing prominent literature about acculturation and global mindset, Vuong and Napier [36] and Vuong [37] developed the concept of “mindsponge” into the mindsponge mechanism.

The mechanism was first proposed by Vuong and Napier [36] to explain “why and how professionals and managers could replace the cultural values they have grown up with those they have absorbed following education and work in “foreign” settings.” Later, Nguyen et al. [3] suggested that the mindsponge mechanism can be employed to explain and construct theoretical models for complex psychological and behavioral issues.

**Fig. 1:**
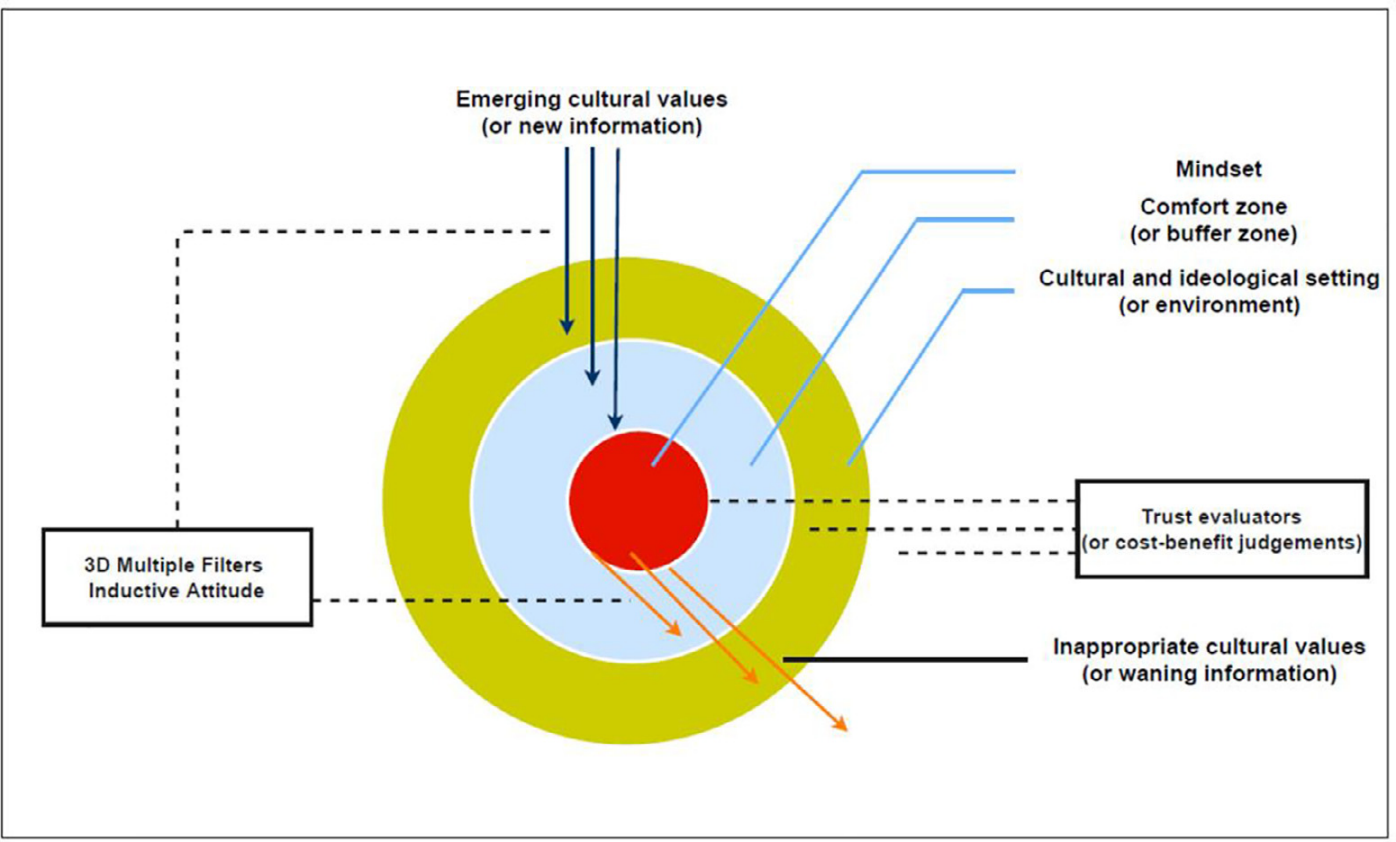
Mindsponge mechanism. The visualization is retrieved from Nguyen, et al. [38] under the Creative Commons Attribution license (CC-BY).

The mindsponge mechanism is dynamic and multiplex, so it is often displayed through a conceptual diagram with a pie shape representing a mind and the surrounding environment (see Fig. 1). The diagram consists of five main components: (1) mindset, (2) comfort zone, (3) multi-filtering system, (4) cultural and ideological setting (or environment), and (5) cultural values (or information).

The outermost part of the pie – the yellow-colored part – demonstrates the cultural and ideological settings (or environment, in general) where the individual is within. The innermost part of the pie – the red-colored nucleus – represents the mindset or a set of core values. Given that culture can be defined as “the set of attitudes, values, beliefs, and behaviors shared by a group of people, but different for each individual, communicated from one generation to the next” [39], the mindset includes but is not limited to cultural values. Highly trusted values or beliefs can also be deemed as core values. Such core values are used as benchmarks, explicit or implicitly, for judging the appropriateness of newly absorbed values (or information) and making decisions or responses. In other words, the mindset greatly influences an individual's perceptions, attitudes, and behaviors. Due to this function of the mindset, it creates a self-protection mechanism for the individual's “self”, which is analogous to the assertion of the self-affirmation theory that “the overall goal of the self-system is to protect an image of its self-integrity, of its moral and adaptive adequacy. When this image of self-integrity is threatened, people respond in such a way as to restore self-worth” [40].

The blue part in the middle of the outermost part and the nucleus is the comfort zone, also called the buffer zone. This zone is constituted by values in the mind that are not core values. It has two fundamental functions. First, any values that want to enter the mindset must pass through the comfort zone, so the comfort zone helps protect the mindset from external shocks when the environment changes swiftly (e.g., cultural shocks). Second, the comfort zone is where the multi-filtering system kicks in to evaluate the appropriateness and usefulness of the newly entering values. Although the filtering process can happen anywhere within the mind, no matter how close it is to the mindset, the white membranes between the yellow/blue and blue/red parts represent the point of evaluation or filtering (for easier interpretation). The closer the values are to the mindset, the stricter the evaluation or filtering will be.

The multi-filtering system has two basic functions: integration and differentiation of information [41]. When the information from the environment enters the mind, it is treated in two ways. If the information is compatible with the core values (or mindset), it will be synthesized and incorporated through integration. Suppose the emerging information differs from the existing information (or values). In that case, the difference will be measured through differentiation to assess the cost and benefit of accepting or rejecting the emerging values (or replacing existing ones with new ones).

The multi-filtering system is driven by the 3D (triple-discipline) filter [42], the notion of inductive attitude [43], and trust evaluators [44,45]. The 3D filter refers to disciplined processes that evaluate, connect, compare, and imagine emerging and existing values to create useful and ready-to-use insights. The emerging values are new information absorbed from the environment (or out-of-discipline information). In contrast, the existing values are information already stored in memory as trusted knowledge (or within-discipline information). In other words, the 3D filter is a proactive disciplined process of mindsponge filtering that compares foreign information and values to existing trusted values and then decides whether to accept or reject the new values [36]. This filter is operated under the assumption that the individual is aware of their desire(s) or has clear priorities, which aligns with the Theory of Planned Behavior [46].

Pólya [43]’s inductive procedure offers a three-step mechanism to filter information that starts with noticing similarities (or analogies) between new and existing values. Then, such analogies are generalized into conjectures, and the conjectures are eventually tested in a specific context. The inductive attitude is thinking that dares to test and retest certain existing beliefs without fear of being easily contradicted by experience. The testing results are stored in mind and form the “guard of trust” (or trust evaluators). Vuong and Napier [36] suggest that there are at least four levels of trust evaluation:

1) personal qualities and properties;
2) the expectation of future costs and benefits in both the short and long term;
3) ability to verify a value's adaptability to the existing mindset;
4) suitability of generalized values at the philosophical level.

For a piece of information to be received by a person (or be absorbed into a mind), two qualities must be satisfied: availability and accessibility. Availability refers to the information's quality of being available objectively in the environment, while accessibility refers to the mind's quality of being able to access or receptive to the available information. The mind's accessibility to the information is not based solely on the objective availability of the information. Still, it is based on both the objective availability of the information and the perceivable range of the individual simultaneously. According to Nguyen [47], one's perceivable range is “the physical range (or environment) within which a person can see, hear, or become aware of something through the senses.” Due to humans’ limited physical and mental capacity, we cannot perceive, evaluate, and absorb all the information in the perceivable range.

The absorption and ejection of information and values are represented by the arrows moving in and out, respectively. The arrows heading to the nucleus demonstrate the emerging information and values, while the arrows heading out demonstrate the waning values and information no longer compatible with the core values. The in-flows and out-flows are non-stop, continual processes that create the mindsponge mechanism's updating feature. This updating feature helps clarify the distinction between the 3D filter and trust evaluator, which are analogous at first glance because both are related to subjective cost-benefit judgment. To elaborate, the cost-benefit judgment of new values engaging trust evaluators employs the analogous existing values that were formerly absorbed, evaluated, and validated through the inductive procedure. New values aligning with trusted values will be given a “priority pass” that requires less rigorous evaluation to be absorbed into the mindset (and *vice versa* in the case of distrust – a priority for rejection). In contrast, if no existing formerly evaluated and validated values are analogous to the emerging values and information, the emerging values and information must be evaluated carefully as usual by a 3D filter.

Although the mindsponge mechanism is complex and dynamic, it can be summarized into five main principles [2].

1. An information particle must exist within a mind (the subjective world) to be processed by the mind.
2. The information processing mechanism within the mind (the multi-filtering process, or absorption and ejection processes of information and values) is based on the trust evaluator and subjective cost-benefit judgment to maximize the perceived benefits and minimize perceived costs.
3. The multi-filtering process depends on the value system shaped by the mindset (a set of core values).
4. The outputs of conscious and subconscious mental processes (e.g., value system, ideas, thoughts, feelings, behaviors, etc.) are influenced by the values within the mind (mainly by the core values in the mindset).
5. An information particle needs to exist in the environment (objective world) and locate within the perceivable range to be absorbed into the mind (subjective world).

These five principles are also displayed on the logical diagram for better comprehensibility and applicability. More detailed explanations of the mindsponge mechanism can be found here [48].

## Exemplary analysis

In this exemplary analysis, we only concentrate mainly on two core steps of the BMF-based research: (1) the mindsponge-based conceptualization and model construction, and (2) Bayesian analysis and interpretation. For examples of other steps, readers can refer to the following studies [[3], [4], [6], [49], [50], [51], [52]].

Despite the existence of other Bayesian analysis software with more advanced functions, we used the open **bayesvl** R package version 1.0 here due to its visualization power, high pedagogical values, and easy-to-use operation [34,53,54]. The code used to perform Bayesian analysis for the example is available in the Supplementary.

The package has two downloadable versions. Version 0.8.5 can be downloaded directly from The Comprehensive R Archive Network (CRAN). The step-by-step operation protocol of this version is published by *MethodX* [53]. Meanwhile, version 1.0 is publicly available and installable directly from Github (https://github.com/sshpa/bayesvl). The updated version includes the widely available information criterion (WAIC) and Leave-one-out cross-validation (LOO-CV) computation for model comparison and selection, the Posterior Predictive Check, and the visualization function for test quantities. The step-by-step operation protocol of this version is available in the following book chapter [55].

### Mindsponge-based conceptualization and model construction

The example was performed using the dataset of 3,071 Vietnamese entrepreneurs’ decisiveness and perceptions of the likelihood of success/continuity [56]. The dataset can be accessed and retrieved from the following URL: https://data.mendeley.com/datasets/kbrtrf6hh4/2.

Creativity is a key dimension in defining strategic entrepreneurship, so it is essential to understand the factors that can predict a new firm's high creativity level. Also, the dataset used in this example offers variable *X14.inno*, which helps measure the entrepreneurs’ self-evaluated creativeness of product/services/business model. Therefore, our research question is:

• What are factors that can predict Vietnamese entrepreneurs’ self-evaluated creativity level?

We can employ several principles indicated by the mindsponge mechanism to answer this question. According to the mindsponge mechanism, creative products/services/business models are deemed as the output of an entrepreneur's mental processes, so they are influenced by the information previously absorbed into their minds. This assumption is aligned with former studies suggesting that absorbing new out-of-discipline insights and ideas (e.g., from other people) is likely to help improve creativity [42,57]. These insights and ideas also include other people's failures. Therefore, it is plausible to hypothesize that the more failures entrepreneurs learn from others, the more creative outcomes they can generate.

However, the information absorption process is contingent on the mindset. Specifically, suppose the entrepreneur has a proactive mindset to absorb new information from other people's failures (even if they conflict with their existing core values) and eventually transform their core values. In that case, the entrepreneur will be more likely to absorb out-of-comfort-zone values and minimize their self-affirmation bias, which may improve their creativity level. On the contrary, if they are unwilling to change and reject contradicting information, they will be less likely to absorb out-of-comfort-zone values and, thus, less likely to generate creative outcomes, even though they are accessible to information regarding other people's failures. Thus, our second hypothesis is that the association between entrepreneurs’ access to other people's failures and their creativity level is positively moderated by the entrepreneurs’ openness to change.

To test the assumptions above, Model 1 is constructed as follows:

(1) EvaluatedCreativity∼α+ExternalInfor+ExternalInfor*TransMind

From the dataset, we selected variable *X10.failurel* and modified it to variable *ExternalInfor*, which represents the degree of entrepreneurs’ accessibility to information about other people's failures. For measuring the openness to change of entrepreneurs, we employed variable *X19.msponge* and modified it into TransMind. More details of Model 1’s outcome and predictor variables are shown in Table 1.

**Table 1:** Description of outcome and predictor variables.

Modified variables	Original variables (in the dataset)	Meaning	Type of variable	Value
*EvaluatedCreativity*	*X14.inno*	Self-evaluation of creativeness of product/services/business model	Numerical	1 = not at all 2 = hopefully 3 = somewhat creative 4 = creative
*ExternalInfor*	*X10.failurel*	Whether the entrepreneur learns from other peoples׳ failures	Continuous	1 = no need 2 = exploring few noteworthy cases 3 = careful study
*TransMind*	*X19.msponge*	Entrepreneurs’ efforts to transform ways of thinking, acting and beliefs	Continuous	1 = none 2 = negligible 3 = some aspects 4 = strong

### Bayesian analysis

We employed the **bayesvl** package to construct and fit Model 1 using both informative and uninformative priors. For incorporating informative priors into the model, we set prior distributions of both parameters *ExternalInfor* and ExternalInfor*TransMind as normal distributions with mean values at 1 and standard deviations at 0.5 to reflect our belief in the positive predictions of both variables. This belief is rationalized by the theoretical support of the mindsponge mechanism described above.

Before interpreting the posterior results, two fundamental steps need to be taken. First, the model's goodness-of-fit with the data has to be checked to see whether the model is well-specified employing the Pareto smoothed importance-sampling leave-one-out cross-validation (PSIS-LOO) approach. In this case, the *k*-values of Model 1 are all well below 0.5 (see Fig. 3), indicating that the constructed model is well specified.

Next, the convergence of the model's simulated samples needs to be diagnosed to make sure the Markov chain central limit theorem is held. The diagnosis can be conducted in two ways:

1. *Using two standard diagnostics of MCMC simulation: n_eff* and *Rhat*. The *n_eff* value indicates the number of iterative samples that are not autocorrelated during the stochastic simulation process. Generally, it is accepted that if the *n_eff* value is greater than 1000, the Markov chains are convergent, and the effective samples are enough for accurate inference [23]. *Rhat* value – also known as the Gelman shrink factor and the potential scale reduction factor, shows the convergence of the logarithm [58]. The model is deemed convergent if the Rhat value is equal to 1.
2. *Using visual diagnostics*. Although it is possible to diagnose the convergence using *n_eff* and *Rhat* values, many Bayesian statisticians consider the trace plot a ‘must have’ for diagnosing the convergence. The trace plot illustrates the MCMC sample values after each successive iteration along the chain. The plot's *y*-axis demonstrates the coefficient's value, while the *x*-axis demonstrates the number of iterations of the Markov process. If the chains are good-mixing and stationary around an equilibrium, the Markov chains can be deemed well converged. Besides the trace plot, the Gelman and autocorrelation plots are also helpful diagnostic tools. The Gelman plot examines whether the Gelman factor drops rapidly to 1 before the warmup period completes. The simulated samples can be deemed satisfactorily convergent if the factor drops to 1 before the 2000th iteration. Regarding the autocorrelation plot, it illustrates the degree of correlation between MCMC samples separated by different lags. For example, a lag of 0 represents the degree of correlation between each MCMC sample and itself (obviously, this will be a correlation of (1). Four lines in the autocorrelation plot correspond to the autocorrelation levels among iterative samples generated by four Markov chains, respectively. If the autocorrelation levels among iterative samples drop to 0 after some finite lags, the iterative samples are considered independent, and the Markov chains are deemed convergent.

As can be seen from Table 2, all parameters’ *n_eff* values are larger than 1000, and *Rhat* values are equal to 1. This suggests that Model 1’s simulated samples are well convergent. The convergence is also confirmed by the trace plots (Fig. 4), Gelman plots (Fig. 5), and autocorrelation plots (Fig. 6).

**Table 2:** Simulated posteriors.

Parameters	Informative priors				Uninformative priors			
	Mean	Standard deviation	n_eff	Rhat	Mean	Standard deviation	n_eff	Rhat
*Constant*	1.84	0.06	6128	1	1.85	0.06	5325	1
*ExternalInfor*	0.22	0.04	4882	1	0.21	0.04	4212	1
ExternalInfor*TransMind	0.05	0.01	6310	1	0.05	0.01	5243	1

When all the diagnostics indicate the good convergence of Model 1, it is possible to interpret the posterior results. Table 2 shows that both *ExternalInfor* and ExternalInfor*TransMind positively predict *EvaluatedCreativity*. The predictions are also highly reliable as the posterior distributions’ standard deviations are much smaller than the mean values ($\mu_{ExternalInfor}=0.22$ and $\sigma_{ExternalInfor}=0.04$; $\mu_{ExternalInfor*TransMind}=0.05$ and $\sigma_{ExternalInfor*TransMind}=0.01$). Visually, the 90% Highest Posterior Density Intervals (HPDIs) of both parameters lie entirely on the positive side of the *x-*axis, suggesting that the predictions of *ExternalInfor* and ExternalInfor*TransMind against *EvaluatedCreativity* have at least a 90% probability of being positive (see Fig. 7). The posterior results remain unchanged, even when we fitted the model again using uninformative priors. This signals good robustness of the model's estimation.

**Fig. 2:**
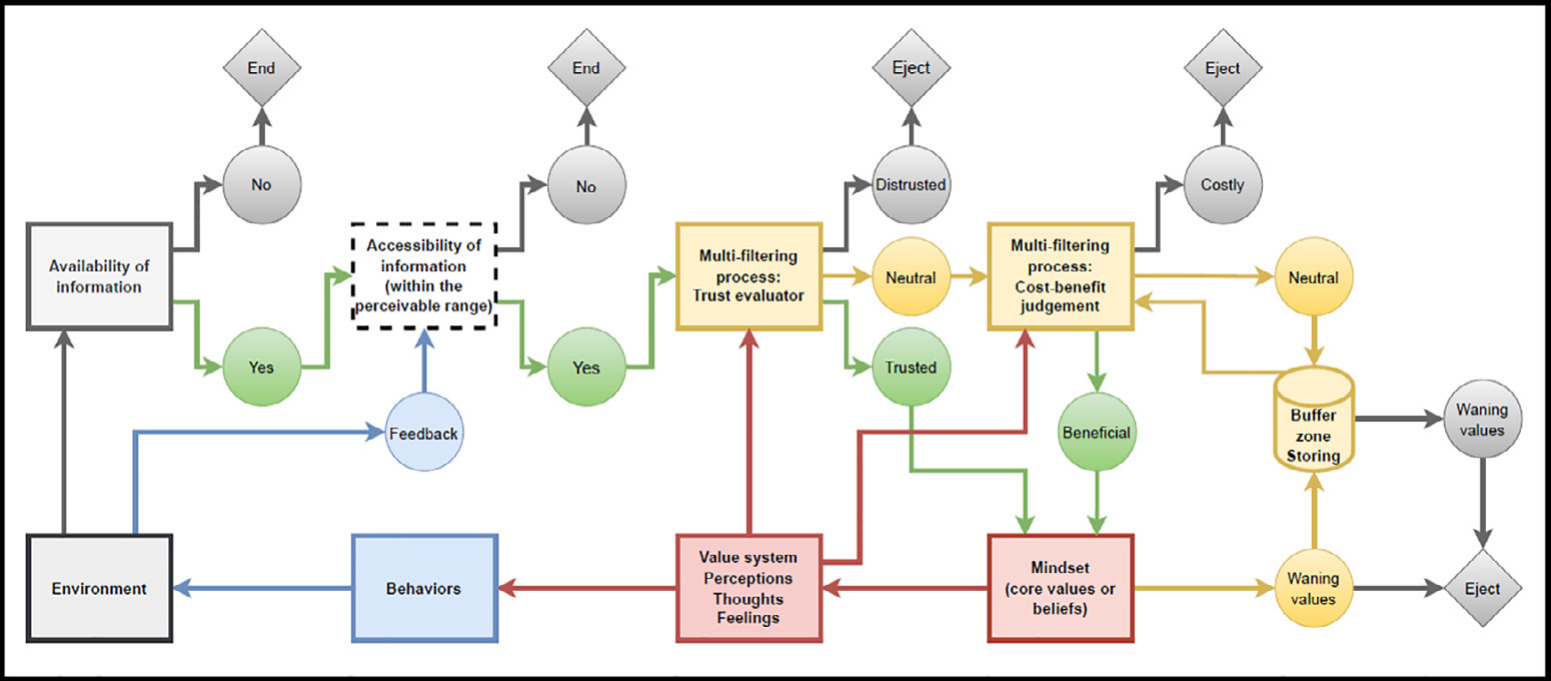
The logical diagram of the mindsponge mechanism's five main principles, directly borrowed from Nguyen and Vuong [49].

The results confirm previous studies about the positive impact of out-of-discipline insights and ideas (e.g., from other people) on the self-evaluated creativeness of product/services/business model [42,57] and validate the mindsponge-based assumptions on information accessibility and information filtering process. Further studies can be performed to investigate other aspects of the mindsponge mechanism, as shown in Fig. 2, such as the influence of mindset, value system, perceptions, and types of information on self-evaluated creativeness.

**Fig. 3:**
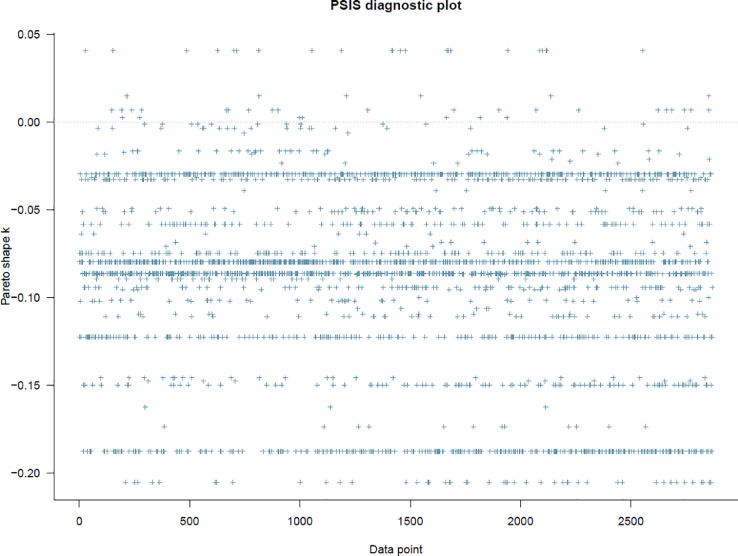
PSIS diagnostic plot.

**Fig. 4:**
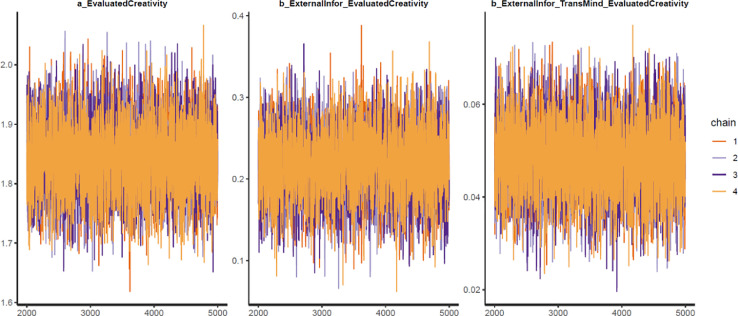
Trace plots.

**Fig. 5:**
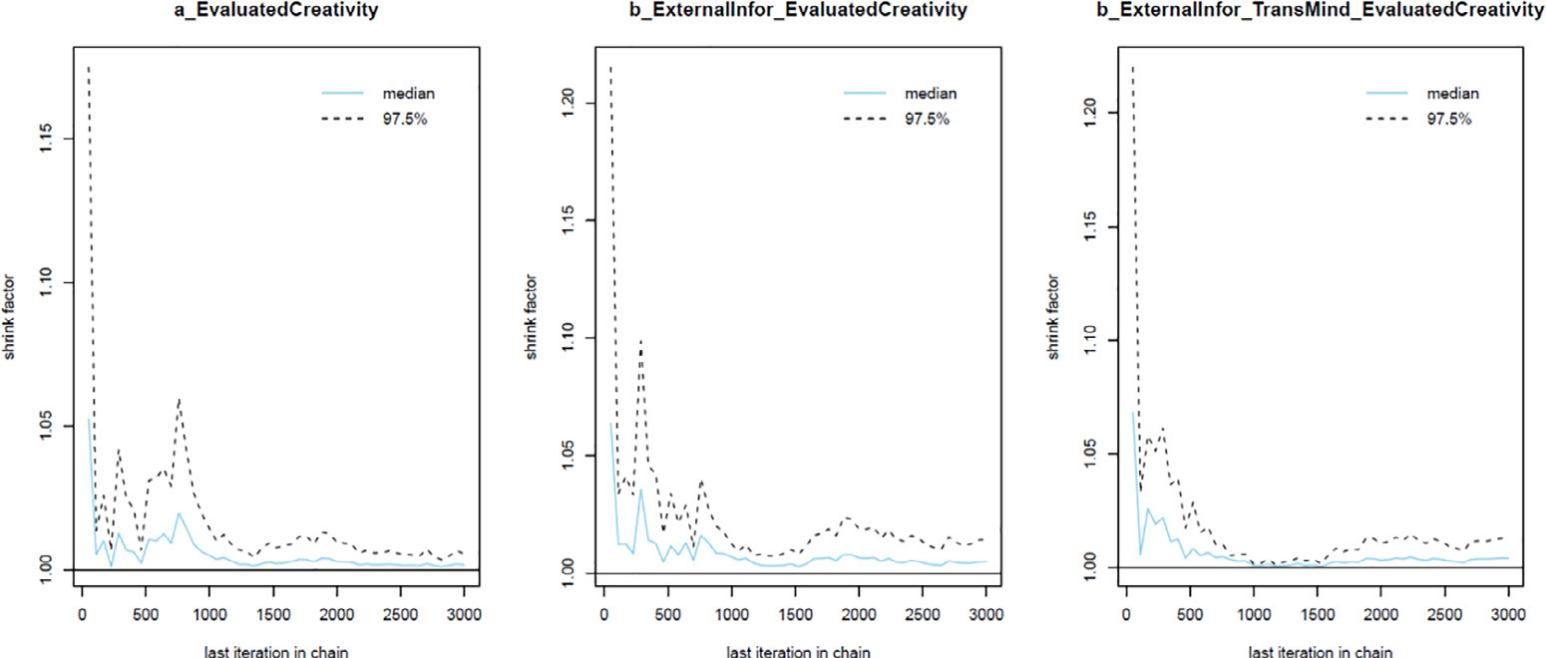
Gelman plots.

**Fig. 6:**
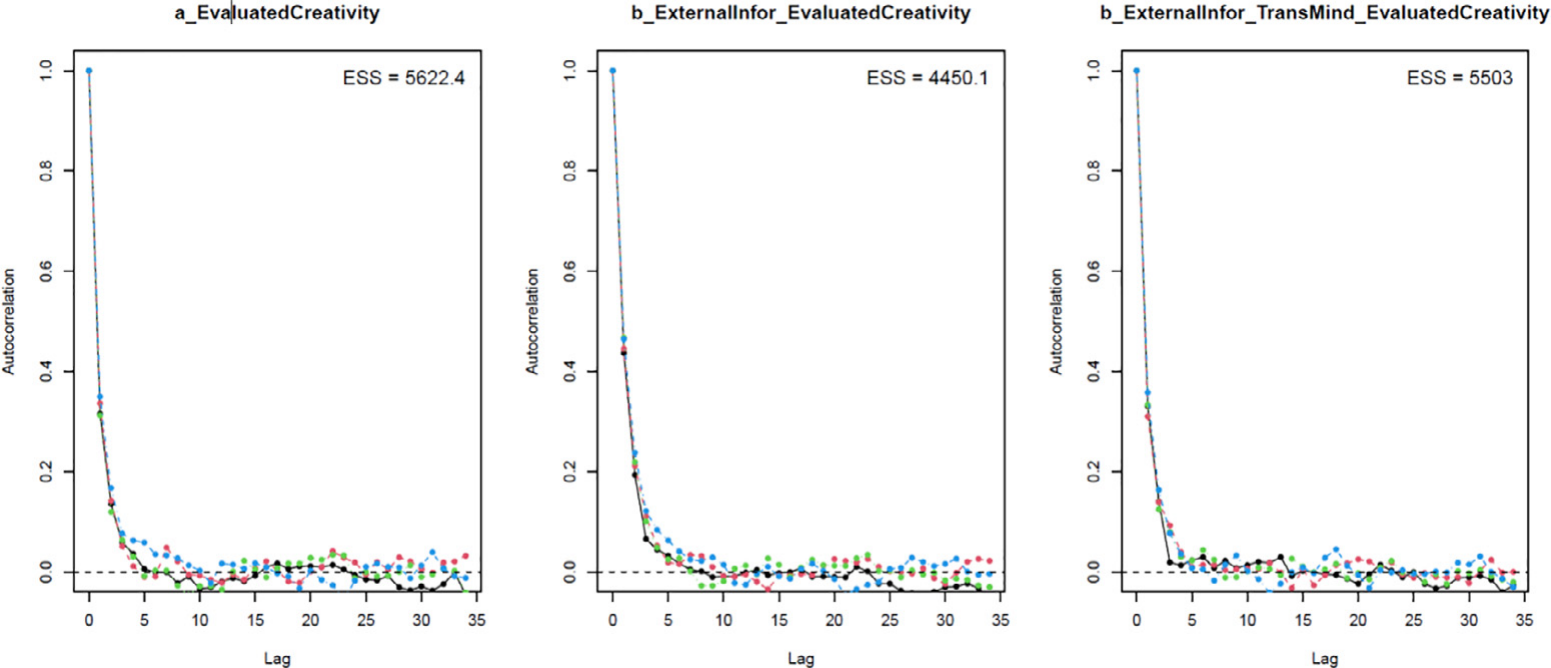
Autocorrelation plots.

**Fig. 7:**
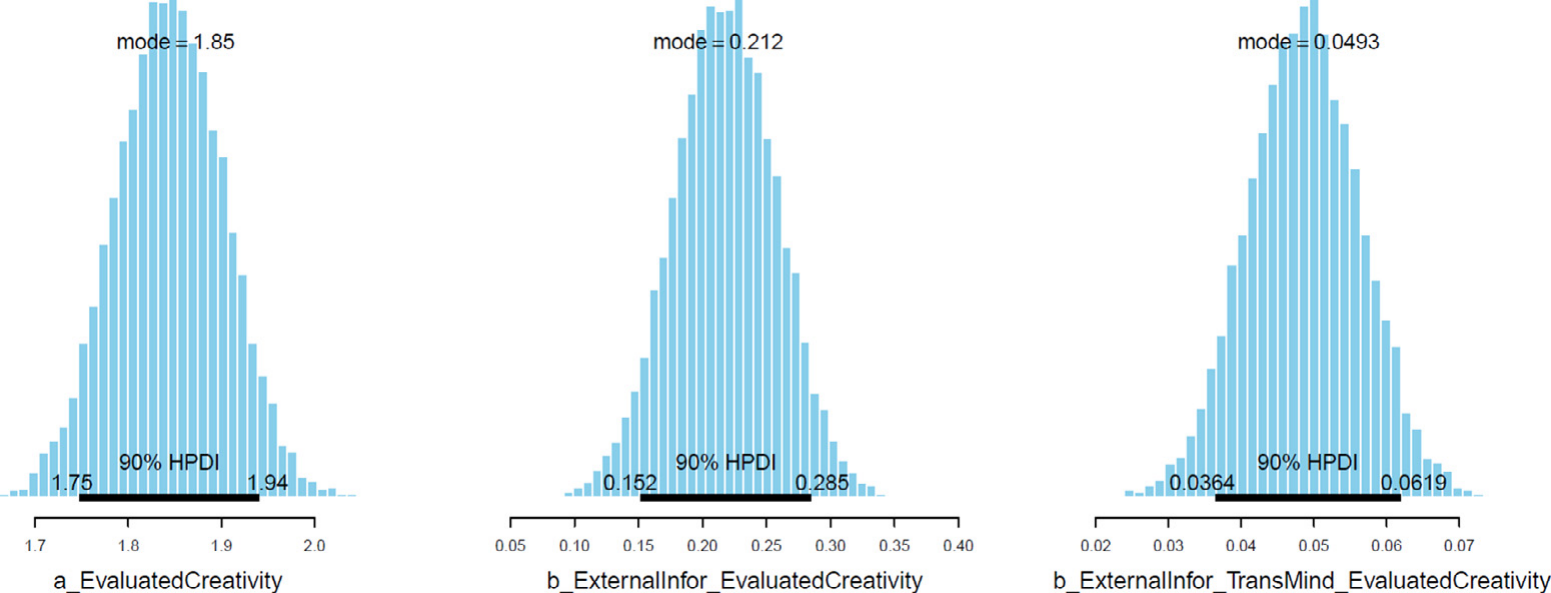
Parameters’ posterior distributions with HPDI at 90%.

## Final remarks

BMF analytics is a useful tool for researchers to innovate and generate originality. As the mindsponge mechanism is an open information-processing system for thinking (not bounded by any specific discipline or context), researchers can apply the mechanism to develop their own approaches to tackle different problems in different contexts or even make their own theories for explaining human and social nature. Then, the Bayesian inference, with its great fit with the mindsponge mechanism, will support researchers in validating their proposed ideas and theories.

Our understanding of human beings at the societal level has been advanced by many great theories and frameworks, such as Maslow's Hierarchy of Needs [59,60], Hofstede's cultural dimensions [61], and Porter's Generic Strategies model [62], etc. Although the mindsponge mechanism has certain advantages in studying humans due to its updating features and flexibility derived from the information-based approach, it does not intend to replace other well-known theories and frameworks but to complement and enrich them, especially when humans have to change and adapt to crises, the dynamic Internet era, and technological advancement [63], [64], [65], [66], [67]. One typical example is the BMF-based study on the suicidal ideation mechanism [3]. The study did not reject other prominent theories in the field, like the Interpersonal Theory of Suicide [68] and the Integrated Motivational–Volitional Model [69], but complemented them by indicating additional conditions that can lead to the emergence of suicidal ideation, besides thwarted belongingness.

Moreover, the mindsponge mechanism is an information-processing framework. From a metaphysical standpoint, anything can be examined in terms of information [70]. We make sense of the world based on the information we receive, and all science disciplines need to work with information [71], including human consciousness [72]. Therefore, BMF analytics offers researchers great flexibility to research multiple and interdisciplinary topics in various fields.

BMF analytics is a method combining the conceptual formulation power of the mindsponge mechanism and inferential power of the Bayesian analysis, but researchers can capitalize either the mindsponge mechanism or Bayesian analysis separately for conducting research. High-quality studies using Bayesian analysis are prevalent, so readers can easily meet one when searching the literature. The following works are typical instances of conceptual and theoretical publications that use the mindsponge mechanism [73], [74], [75], [76], [77].

Last but not least, because the method emphasizes effectiveness, efficiency, and transparency, resources connected with BMF-based studies (e.g., data, software, code) are usually open, allowing researchers to refer to, reproduce, and examine the investigated issue further. Here are some examples [3], [4], [5], [6], [7]. We also encourage researchers using BMF analytics to be open about their data and code since this will improve the scientific integrity of their studies and foster knowledge exchange in academia.

## CRediT authorship contribution statement

**Minh-Hoang Nguyen:** Conceptualization, Methodology, Software, Writing – original draft. **Viet-Phuong La:** Validation, Data curation, Software, Writing – original draft. **Tam-Tri Le:** Visualization, Investigation, Writing – review & editing. **Quan-Hoang Vuong:** Supervision, Conceptualization, Methodology, Software, Validation.

## Declaration of Competing Interest

The authors declare that they have no known competing financial interests or personal relationships that could have appeared to influence the work reported in this paper.

## Data Availability

I have shared link to the data in the manuscript.

## Appendix Supplementary materials

Image, application 1

Image, application 2
